# A conditional transgenic reporter of presynaptic terminals reveals novel features of the mouse corticospinal tract

**DOI:** 10.3389/fnana.2013.00050

**Published:** 2014-01-07

**Authors:** Pasquale D’Acunzo, Aurora Badaloni, Mattia Ferro, Maddalena Ripamonti, Vincenzo Zimarino, Antonio Malgaroli, G. Giacomo Consalez

**Affiliations:** ^1^Division of Neuroscience, San Raffaele Scientific InstituteMilan, Italy; ^2^Università Vita-Salute San RaffaeleMilan, Italy; ^3^MTM S.r.l.Milan, Italy

**Keywords:** amyotrophic lateral sclerosis, synapses, transgenic mice, GFP labeled, presynaptic terminals, reporter mouse, neurodegenerative, axons

## Abstract

In many neurodegenerative disorders, including amyotrophic lateral sclerosis (ALS), synaptic alterations precede the demise of the neuronal cell, making synapses a useful vantage point from which to monitor the onset and progression of clinical signs and pathological changes. While murine models of ALS display many features in common with the clinical picture observed in patients, corticospinal tract (CST) involvement is usually less severe in mice than the picture observed in humans. In this paper we describe the characterization of a new conditional transgenic line obtained by targeted integration of a GFP-VAMP2 fusion gene into the Rosa26 locus, and devised to permit the detection of genetically defined presynaptic terminals in wild type mice and murine models of neural disorders. This reporter molecule is selectively enriched in presynaptic boutons, significantly reducing the background signal produced by fibers of passage. The specific features of this reporter line allow us to strongly support the view that murine CST terminals give rise to very few direct contacts with spinal motor neurons. Moreover, the evidence described here reveals the existence of previously uncharacterized, putative direct connections between CST presynaptic boutons and Renshaw neurons in the spinal cord. These results constitute a proof of concept for the potential application of this indicator line to morphological analyses of wild type and diseased synapses.

## INTRODUCTION

Numerous murine models of neurodegenerative diseases exist and, in many of them, synaptic alterations predate the demise of the neuronal cell body and can be used as a predictor of disease onset and progression (reviewed in Conforti et al., 2007). In amyotrophic lateral sclerosis (ALS) the first subcellular damage detected at early stages of the disease is axonal and synaptic (Fischer et al., 2004). More broadly, axon terminals provide an early vantage point for the study of neurodegenerative disorders.

In animal models, the gold standard for the study of neuroanatomical and functional connections in the nervous system is represented by the local injection of molecules called tracers which are transported or diffuse along axons in a retrograde or anterograde direction. Depending on the nature of the selected molecule, these substances can travel *in vivo* from the cell soma to the axon terminal or vice versa, and are visualized thanks to (immuno) histochemical techniques (Zaborszky et al., 2006). While these approaches guarantee cellular resolution, sensitivity and stability, several pitfalls in their use remain. First, they require surgical expertise and, in some cases, the use of complicated procedures, introducing variables and a lack of reproducibility related to the operator’s experience. Second, slight variations in the location of the injected area or in the amount of tracer can lead to considerable differences between experiments, particularly in high resolution experiments and in small animals such as mice. Third and most important, signals often come from unwanted sources; indeed, some anterograde tracers also act as retrograde tracers in certain cases (Reiner et al., 2000); likewise, artifactual tracing can occur if unrelated fibers of passage take up the tracer form their neighbors, e.g., through pinocytosis (Jiang et al., 1993). Finally, all tracing methods per se give information on the position of cells or terminals, but do not provide any cues as to the molecular identity of the corresponding neurons. Thus, the analysis of murine models of neurodegenerative disorders would benefit from the availability of presynaptic terminal markers, particularly transgenic (Tg) reporters permitting the selective detection of genetically defined subsets of synaptic boutons. Cre-activated anterograde reporters make it possible to study both normal development and developmental defects or degenerative changes affecting specific axons and their terminations. Several genetic “tracers” are available to label axons (Bareyre et al., 2005) and circuits, the latter through the Tg expression of trans-synaptic proteins (Braz et al., 2002; Lo and Anderson, 2011). While other reporters already exist, they are usually non-selective, making it difficult to distinguish between presynaptic compartments and axons in transit through a given territory.

In the present paper we describe the generation and characterization of a Cre-activated reporter devised to permit the selective detection of genetically defined presynaptic terminals in murine models of human CNS disorders, with a low axonal background. In addition, we validate our murine model to study the distribution and connections of corticospinal tract (CST) terminations in the spinal cord, an application relevant to the analysis of murine models of motor neuron diseases.

## MATERIALS AND METHODS

### GENERATION OF THE *Rosa26^EGFP-VAMP2^* MOUSE STRAIN

**FIGURE 1:**
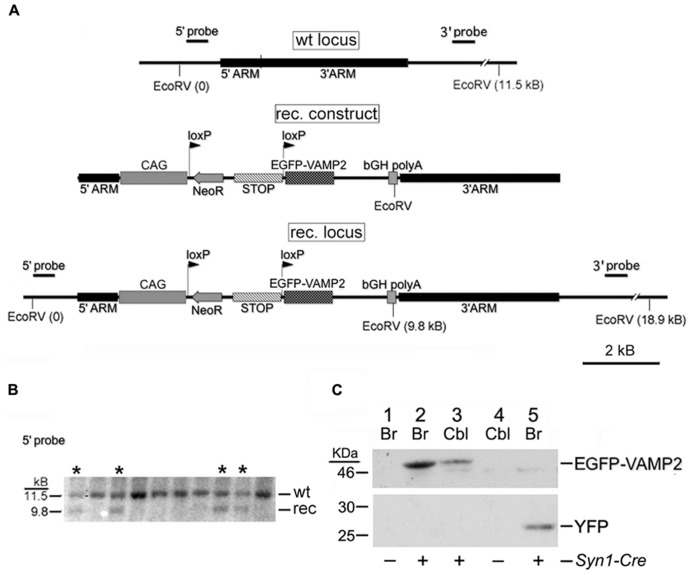
**Generation of the *Rosa26^EGFP-VAMP2^* line. (A)** Schematic representation of the wt *Rosa26* locus, recombinant (rec.) construct and rec. locus obtained by homologous recombination within the 5′ and 3′ homology arms. See Section “Results” for a description of the rec. construct. CAG, chicken β-actin promoter and CMV enhancer. bGH, bovine growth hormone. **(B)** Southern analysis of ES cell clones using the 5′ probe sketched in **(A)**. Lanes marked by asterisks (*) correspond to recombinant clones (note 9.8 kb band). **(C)** Western blot immunostained with an anti-GFP Ab. Adult forebrain (Br) and cerebellar (Cbl) lysates were gel-separated as indicated. Lanes 1–4 contain lysates from *Rosa26^EGFP-VAMP2/+^* mice, revealing a 49 kDa band corresponding to the size of the EGFP-VAMP2 fusion protein; lane 5 contains lysates from *Rosa26^YFP/+^* mice (27 kDa). Lanes 1 and 4 are from *Syn1-Cre* negative mice; Lanes 2, 3, and 5 are from *Syn1-Cre* positive mice.

The DNA fragment coding for EGFP-VAMP2 was first inserted into a plasmid for homologous recombination downstream of a floxed translation/transcription STOP cassette, using standard cloning techniques. From the 5′ to the 3′ the plasmid encompassed a 5′ homology arm for *Rosa26*, the CAG promoter/enhancer (CMV enhancer + β-actin promoter), the STOP cassette flanked by loxP sites, the EGFP-VAMP2 fusion protein, the bovine growth hormone polyadenylation site (bGH pA) and a 3′ homology arm for *Rosa26* (**Figure 1**). A minigene for G418 (neomycin) resistance (Neo^R^) was also inserted within the two loxP sequences, while a suicide gene for negative selection (diphtheria toxin gene) was introduced past the 3′ homology arms. The plasmid was electroporated into murine ES cells, which were cultured in the presence of neomycin. Surviving clones were genotyped by Southern blotting; briefly, after a complete digestion with EcoRV, genomic DNA fragments were electrophoresed on agarose gel and blotted on a membrane, which was eventually hybridized with two distinct radioactive probes, annealing upstream or downstream of the *Rosa26* 5′ and 3′ homology arm. An EcoRV restriction site is present in the transgene but not in the homology arms, so each probe labeled a shorter band in the recombinant locus than in the wild type one (for the 5′ probe: 9.8 Kb vs. 11.5 Kb; for the 3’probe 9.2 Kb vs. 11.5 Kb). Finally, positive ES cells were injected into SV129/SvJ blastocysts, which in turn were implanted into the uterus of pseudo-pregnant female mice. High percentage chimeric founders were crossed with wild type C57BL/6, and agouti progeny were characterized genetically for germline transmission.

### GENOTYPING

Genotyping was conducted by PCR on genomic DNA obtained from tail biopsy using a standard phenol-chloroform (1:1) extraction protocol. For the *Rosa26* locus, both wild type and knock-in EGFP-VAMP2 and yellow fluorescent protein (YFP) transgenes, we used the conditions and the primers reported by Soriano (1999), F_1_: AAGACCGCGAAGAGTTTGTC, F_2_: AAAGTCGCTCTGAGTTGTTAT, and R: GGAGCGGGAGAAATGGATATG. To detect *Emx1-Cre* positive mice, we used universal primers and conditions for the Cre recombinase as suggested by The Jackson Lab, F: TATATCTTCAGGCGCGCGGT and R: GCAATCCCCAGAAATGCCAG. For *Syn1-Cre* we used primers annealing on the *Synapsin1* putative promoter and on the *Cre* recombinase ORF, F: CCAGCACCAAAGGCGGGC, and R: TGCATCGACCGGTAATGCAG. PCR reagents were purchased from Promega.

### TISSUE LYSATES AND WESTERN BLOTTING

*Syn1-Cre+Rosa26^EGFP-VAMP2/EGFP-VAMP2^* and *Syn1-Cre*+* Rosa26^YFP/+^* (positive control) mice were sacrificed, the telencephalon and cerebellum were dissected and immediately plunged into ice cold RIPA buffer with protease inhibitors (1:10 w/v). Tissues were grossly disrupted with a glass potter and homogenized in a small syringe. The samples were sonicated and centrifuged. Supernatants containing proteins were quantified by the BCA assay (Pierce). Proteins (50 μg) were separated through a 12% SDS-polyacrylamide gel and transferred onto a PVDF membrane, as described (Gallagher et al., 2008). The membrane was incubated overnight at 4°C with a rabbit α-GFP (Invitrogen) primary antibody (1:500) and with a goat α-rabbit HRP-conjugated secondary antibody (Bio-Rad) 1:40,000 in 5% w/v non-fat dry milk/TBST. The chemiluminescent reaction was conducted according to recommendations (Pierce).

### TISSUE PROCESSING FOR IMMUNODETECTION

Adult mice were anesthetized with Avertin (0.2 ml/10 g body weight), and transcardially perfused with 4% paraformaldehyde (PFA) in PBS 1X. Brains and spinal cords were post-fixed o/n at 4°C in PFA. Tissues were sunk at 4°C in 30% sucrose. Finally, they were embedded within OCT (Bioptica) and sectioned on a cryotome (16 μm for immunofluorescence and 20 μm for immunohistochemistry).

### IMMUNOFLUORESCENCE

Slides were washed in PBS and blocked/permeabilized in blocking solution (10% Goat Serum, 0,3% Triton X-100 in PBS 1X) for 1 h, then incubated overnight at 4°C with the proper primary antibody: rabbit α-GFP (Invitrogen) 1:500, mouse α-NeuN (Chemicon) 1:300, mouse α-CaBP (Swant) 1:1000 in blocking solution. After several washes, sections were treated for 2 h with secondary antibodies (Goat α-rabbit Molecular Probes Alexa Fluor 488; Goat α-mouse Alexa Fluor 546) 1:1000 in blocking solution. Nuclei were counterstained with Hoechst 1:5000 in PBS for 5 min. To perform immunofluorescence on *Rosa26^EGFP-VAMP2^* slides, we used a Tyramide Signal Amplification (TSA) Kit (Perkin Elmer) to detect EGFP-VAMP2 fusion protein. Briefly, slides were treated with 0,3% H_2_0_2_ in TN buffer for 1 h, blocked/permeabilized for 1 h in TNB solution (0,5% w/v Casein, 0,3% Triton X-100 in TN buffer) then incubated overnight at 4°C with the rabbit α-GFP primary antibody 1:4000 in TNB. Subsequently, slides were washed in TNT buffer (0,1% Tween20 in TN buffer), incubated for 1 h with a biotin-conjugated goat α-rabbit secondary antibody (VectaStain) 1:200 in TNB, washed again, treated for 30 min with HRP-streptavidin (supplied with the kit; 1:150 in TNB) and processed with the tyramide solution for 10 min. Other markers on the same sections were revealed using protocols for ordinary immunofluorescence, as explained above. We used mouse α-synaptotagmin1 1:500 (Synaptic Systems), mouse α-CaBP 1:300 (Swant) and goat α-Choline Acetyltransferase (ChAT) 1:50 (Choline O-acetyltransferase, Millipore) to label presynaptic terminals, Renshaw cells, and motor neurons, respectively. Rabbit VGluT2 1:500 (Vesicular Glutamate Transporter 2, Synaptic Systems) was also used to label cerebellar mossy fibers terminals. After several washes, sections were treated for 2 h with fluoresceinated secondary antibodies 1:1000 in blocking solution. Nuclei were counterstained with Hoechst 1:5000 in PBS for 5 min.

### IMMUNOPEROXIDASE STAINING – DAB AMPLIFICATION

*Emx1-Cre*+* Rosa26^GFP-VAMP2+^*mice brain sections were treated with 0,3% H_2_0_2_ in PBS for 30 min, permeabilized in P-solution (1.72 M sucrose, 50 mM NaCl, 3 mM MgCl_2_, 20 mM Hepes, 0,5% Triton X-100) for 10 min, blocked 1 h in a goat serum-based blocking solution then incubated overnight at 4°C with the rabbit α-GFP primary antibody 1:500 in blocking solution. Subsequently, slides were treated with a biotin-conjugated goat α-rabbit secondary antibody 1:200 in blocking solution for 2 h. Several washes followed, then a streptavidin-HRP containing solution (ABC solution, VectaStain) for 30 min. The chromogenic solution contained 0.3 mg/mL diaminobenzidine (DAB, Sigma), 0.1% Tween, 0.03% H_2_0_2_ in PBS. The reaction was blocked with 0.001% NaN_3_ in PBS. Nuclei were counterstained as described above.

### NISSL STAINING

Slides were washed in PBS, dehydrated with a rising ethanol scale (50% for 3 min, 70% for 3 min, 95% for 3 min, pure EtOH for 1 min), submerged in an ethanol/chloroform (1:1) solution for 20 min, rehydrated with a descending ethanol scale (95% for 5 min, 70% for 10 min, 50% for 10 min, pure ddH_2_0 for 5 min), stained with cresyl violet acetate for 15 min, post-fixed with 4% cold PFA for 15 min, dehydrated again and treated with a de-differentiation solution (two drops of glacial acetic acid in 100 mL of 95% EtOH). Finally, they were rinsed in xylene and mounted with a xylene mounting gel.

### IMAGE ACQUISITION AND PROCESSING

Slides were examined on a Leica Confocal (20x-40x-63x) and a Zeiss Axioplan 2 (5x, 10x, 20x) epifluorescence microscope. In some cases, images were further magnified digitally. Minor adjustments in term of contrast or brightness were made with Adobe Photoshop CS4 version 11.0.2. (Adobe Systems). This program was also used for merging and collages. Spinal cord stacks were processed using the ImageJ software (NIH).

## RESULTS

### GENERATION OF *Rosa26^EGFP-VAMP2^* KNOCK-IN MICE

We have developed a new *Cre*-inducible presynaptic reporter consisting of the presynaptic protein VAMP2 fused N-terminally to EGFP.

The goal of this project was to produce an indicator/reporter line expressing a fusion protein targeted to the secretory vesicle wall, and to achieve a moderate expression level so as to avoid overexpression artifacts that are common with chimeric proteins, often due to defective subcellular trafficking of unfolded proteins.

The chimeric molecule was constructed as follows: starting from a 2 Kb rat *Vamp2* cDNA (Elferink et al., 1989), the CDS was modified in order to generate an N-term fusion to EGFP and a C-term fusion to a spacer peptide derived from the ectodomain of TfR (Grote et al., 1995) followed by a Myc tag. The resulting doubly fused CDS was contained within a 2.7 kb fragment comprising 1.5 Kb of 3′ UTR sequences from rat *Vamp2.*

Our “genetic tracer” (sketched in **Figure 1A**) was inserted by homologous recombination into the *Rosa26* locus, which is insensitive to epigenetic silencing and ensures stable, ubiquitous transgene expression (Mao et al., 2001; Srinivas et al., 2001). The transgene is preceded by a floxed transcriptional/translational stop cassette (**Figure 1A**). In basal conditions, the protein is not produced; in *Cre*-expressing cells, or cells in which a Cre-human estrogen receptor fusion protein is activated post-translationally with tamoxifen, the stop cassette is excised and the transgene is switched on. A construct, containing homology arms for the *Rosa26* locus, a G418 resistance minigene (Neo) and a negative selection gene (encoding the Diphtheria toxin) was used to electroporate murine ES cells. G418-resistant ES clones were genotyped by Southern blotting (**Figure 1B**), to identify homologous recombinants. Positive ES cells were injected into SV129/SvJ blastocysts, generating chimeric mice. Tg progeny, heterozygous or homozygous, were viable, healthy, fertile, and of normal size.

*Rosa26^EGFP-VAMP2^* mice were mated with *Syn1-Cre*+*/0* mice to obtain *Syn1-Cre*+*Rosa26^EGFP-VAMP2/+^* littermates. Again, *Rosa26^EGFP-VAMP2/+^*animals expressing the *Syn1-Cre*+*/0* or the other recombinases tested were phenotypically indistinguishable from their littermates. In doubly Tg animals, western blots were immunostained with an EGFP Ab to detect EGFP-VAMP2 in telencephalic and cerebellar protein extracts (**Figure 1C**). Our results revealed a clear specific band at the expected size (49 KDa); no smearing was detected, ruling out protein degradation. We also confirmed that the floxed STOP cassette effectively prevents transcription of the construct, since in *Cre* negative individuals (lanes 1 and 4) no specific bands were detected (note that the band around 46 KDa is non-specific, as it is found in all samples, including the ones that express YFP – lane 5).

### IN *SYN1-Cre+ Rosa26^EGFP-VAMP2/+^* MOUSE CEREBELLA, PONTINE MOSSY FIBER ROSETTES ARE SPECIFICALLY LABELED

**FIGURE 2:**
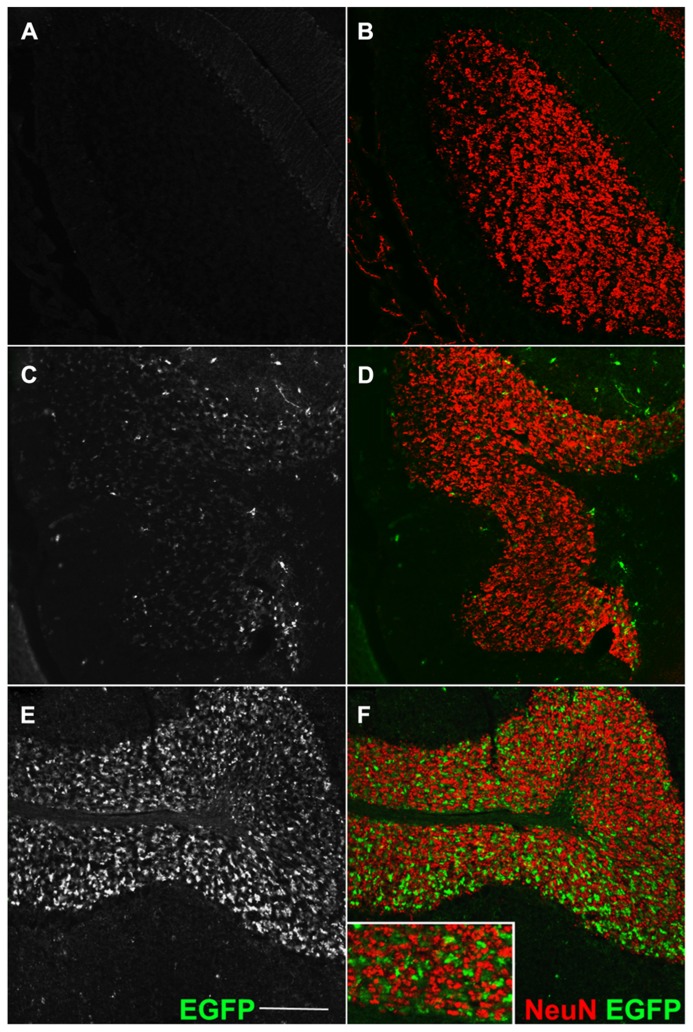
**Transgene expression is entirely Cre-dependent.** Sagittal sections of an adult cerebellum, immunostained for EGFP **(A,C,E)** and counterstained for the GC-marker NeuN. **(A,B)** Double immunofluorescence for GFP and NeuN (granule cells marker) in *Cre*-negative* Rosa26^GFP-VAMP2/+^* mice. Absence of signal confirms the lack of fusion gene transcription in cells which do not express the *Cre* recombinase. **(C–F)** Same immunodetection in *Syn1-Cre*+* Rosa26^GFP-VAMP2/+^* mice. **(C,D)** Lobule I and II; **(E,F)** lobule IX. EGFP+ mossy fiber terminals, originating from Syn1-Cre+ neurons in pontine nuclei (**Figures A1A,B** in Appendix), are particularly abundant in posterior lobules **(E,F)**. Note that EGFP signal (axon terminals) does not overlap with NeuN signal, which decorates the nucleus and cytoplasm of GCs (Leto et al., 2006; Dredge and Jensen, 2011). Scale bar: 150 μm.

**FIGURE 3:**
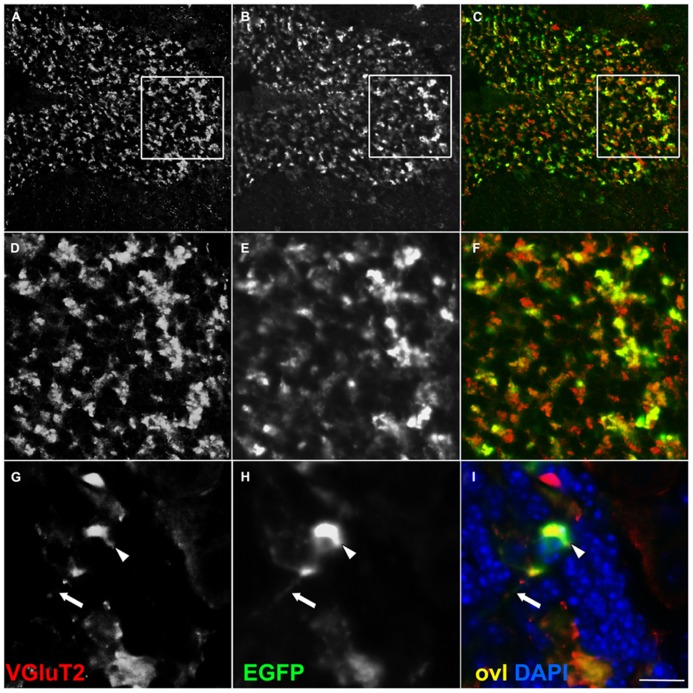
**EGFP decorates presynaptic terminals in the internal granule layer of -Cre *Rosa26^EGFP-VAMP2/+^* mice**. Sagittal sections of an adult cerebellum, immunostained for EGFP **(B,E,H)** and counterstained for the glutamatergic axon terminal VGluT2 **(A,D,G)**, which, in the IGL, labels mossy fiber presynaptic terminals. Overlay (ovl) in (**C,F,I)**. In high magnifications **(G**–**I)** note EGFP+ terminal (arrowhead in **H**) and faintly labeled distal axon (arrow in **H**). Scale bar: **(A**–**C)** 75 μm; **(D**–**F)** 25 μm; **(G**–**I)** 10 μm.

**FIGURE A1:**
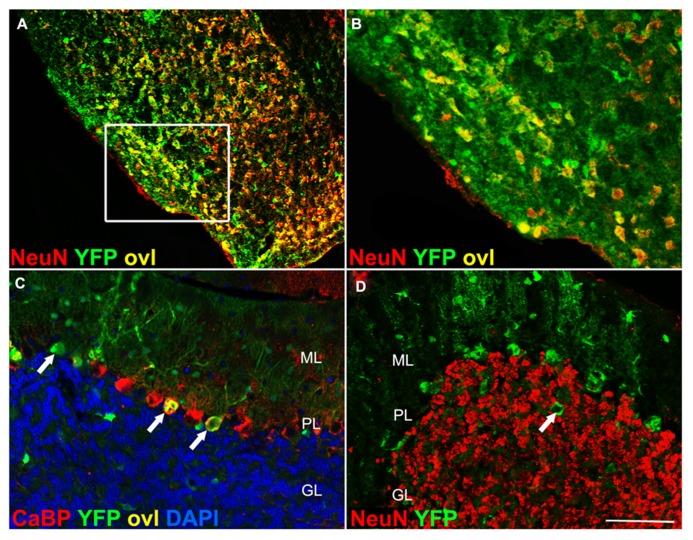
**Syn1-Cre** is expressed in pontine nuclei but not in cerebellar granule cells. **(A)** In *Syn1-Cre Rosa26-YFP* mice, YFP immunoreactivity is detected in pontine nuclei (inset magnification in **B**). In the cerebellum, YFP immunoreactivity (arrows in **C**) is detected in calbindin+ Purkinje cells (arrows in **C**). Large YFP + cell bodies in the granule cell layer (arrow in **D**) are NeuN negative, likely representing Golgi interneurons. CaBP, calbindin; ovl, overlay; ML, molecular layer; PL, Purkinje cell layer; GL, granule cell layer. Scale bar: **(A)** 150 μm; **(B)** 70 μm; **(C,D)** 75 μm.

We investigated whether our fusion protein specifically marks presynaptic terminals *in vivo*. First of all, we analyzed the pontocerebellar tract, which connects mostly the basal pontine nuclei to cerebellar granule cells (GCs) residing in the lateral lobules. We chose this pathway as a proof-of-principle because mossy fibers terminate as large synaptic varicosities (8–13 μm, Wu et al., 1999) with a peculiar “rosette-like” shape which makes them easy to identify. As a strain expressing the *Cre* recombinase at the source but not at the termination of this tract, we utilized the *Syn1-Cre*+*/0* line (Zhu et al., 2001). In these mice, Cre recombinase is expressed only in a subset of synapsin 1+ neurons, possibly due to a positional effect or to the short stretch of promoter used to generate this transgene (Hoesche et al., 1993). *Syn1-Cre*+*/0* mice were mated with *Rosa26^YFP/+^*animals (Srinivas et al., 2001) in order to label *Cre* positive cells. Many basal pontine neurons (NeuN+ cells) were positive for YFP (**Figures A1A,B**). In the cerebellar cortex, the reporter decorated basket cells, stellate cells, and some calbindin (CaBP)+ PCs (**Figure A1C**); conversely, CGs were uniformly negative. Even if scattered large YFP+ cells in the granule cell layer (GL) were detected, they were invariably negative for NeuN (**Figure A1D**), which exclusively decorates GCs in the murine cerebellar cortex (Weyer and Schilling, 2003). Thus, they probably represent internal granule layer (IGL) GABA interneurons (likely Golgi cells). *Syn1-Cre*+*/0* mice were then mated with *Rosa26^EGFP-VAMP2/+^* mice to analyze the spatial distribution of the reporter in sagittal cerebellar sections. We observed a clear signal in the GC layer of lateral hemispheres; this signal did not colocalize with the GC marker NeuN (**Figure 2E**, lobule IX). Interestingly, in the anterior lobe, which does not receive projections from the pons, only a weak, sparse signal was visible (**Figures 2C,D**, lobules I and II). Finally, adult cerebellar sections were stained for EGFP and for the vesicular glutamate transporter VGluT2. In postnatal and adult cerebella, VGluT2 decorates mossy fiber terminals, climbing fiber terminals and a subgroup of unipolar brush cell terminals; as expected, EGFP-VAMP2 and VGluT2 colocalized tightly with presynaptic terminals located in the GC layer (**Figures 3A–F**). Higher magnifications of a single synaptic structure also confirmed the “rosette-like” shape and the expected size (arrowhead in **Figure 3H**). Note that the axonal stem and its collaterals (diameter ~1 μm, Wu et al., 1999) were also weakly labeled (arrow in **Figure 3H**). These results clearly indicate that the reporter is correctly produced and transported from pontine cell somata to their presynaptic terminal in the cerebellum of *Syn1-Cre*+*/0 Rosa26*^EGFP-VAMP2^mice.

### ANATOMICAL CHARACTERIZATION OF CORTICOSPINAL TRACT TERMINATIONS IN *Emx1-Cre+ Rosa26^EGFP-VAMP2/+^* ADULT BRAIN

Next, we investigated the distribution of EGFP+ putative synaptic boutons in the corticopontine (CPT) and CST. The latter is the longest monosynaptic fiber tract in the CNS and degenerates in the course of ALS. In *Emx1-Cre* Tgs, the recombinase is expressed exclusively in glutamatergic neurons and glial cells of the cerebral hemispheres (Gorski et al., 2002), at the origin of the CPT and CST tracts, but not in their target territories, i.e., the brainstem and spinal cord. First, we reproduced published results by breeding *Emx1-Cre* into the *Rosa26^YFP/+^*background, to confirm *Cre* localization in the deep layers of the dorsal pallium, and particularly in the primary motor cortex (M1). As expected, we found a large percentage of YFP+ neurons (**Figure A3**, YFP+ NeuN+ cells).

**FIGURE 4:**
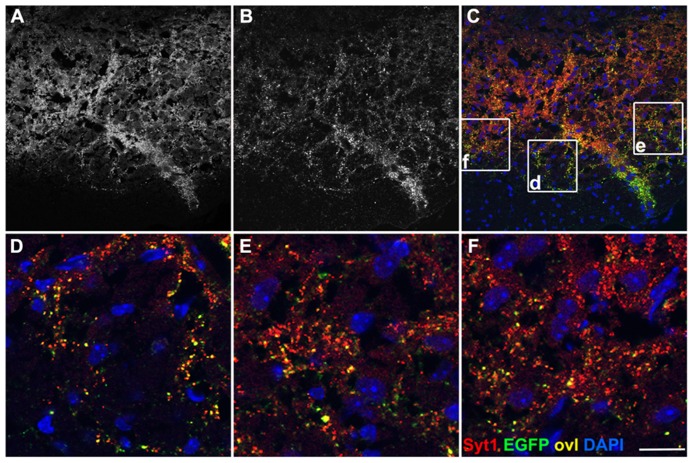
**EGFP decorates presynaptic terminals in the pons of Emx1-Cre *Rosa26^EGFP-VAMP2/+^*.** Sagittal sections of the pontine nuclei, immunostained for EGFP **(B)** and counterstained for the synaptic vesicle protein synaptotagmin1 (Syt1) **(A)**. Overlay in **(C)**. **(D**–**F)** are magnifications of the corresponding insets in **(C)**. Double-positive puncta (yellow, ovl) represent corticopontine axon terminals. Scale bar: **(A**–**C)** 75 μm; **(D**–**F)** 15 μm.

**FIGURE A2:**
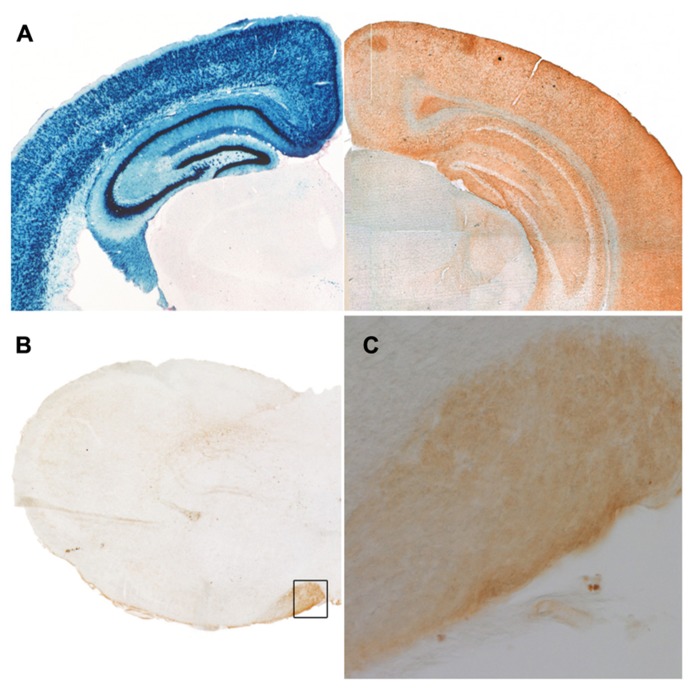
**At low resolution, EGFP-VAMP2 (Maiuscolo) signal is detectable in fiber-rich, cell-body-poor territories.** **(A)** *Left*: LacZ staining of a frontal adult *Emx1-Cre Rosa26*^LacZ/^^+^ mouse forebrain hemisection reveals location of cell bodies (reproduced with permission from Gorski et al., 2002); *right*: similar section from an *Emx1-Cre Rosa26*^EGFP-VAMP2/^^+^ brain immunostained for EGFP reveals complementary distribution of the reporter, which is enriched in cell-poor regions. **(B)** Likewise, in a *medulla oblongata* hemisection, EGFP immunoreactivity is concentrates at the level of the pyramids (inset magnification in **C**).

**FIGURE A3:**
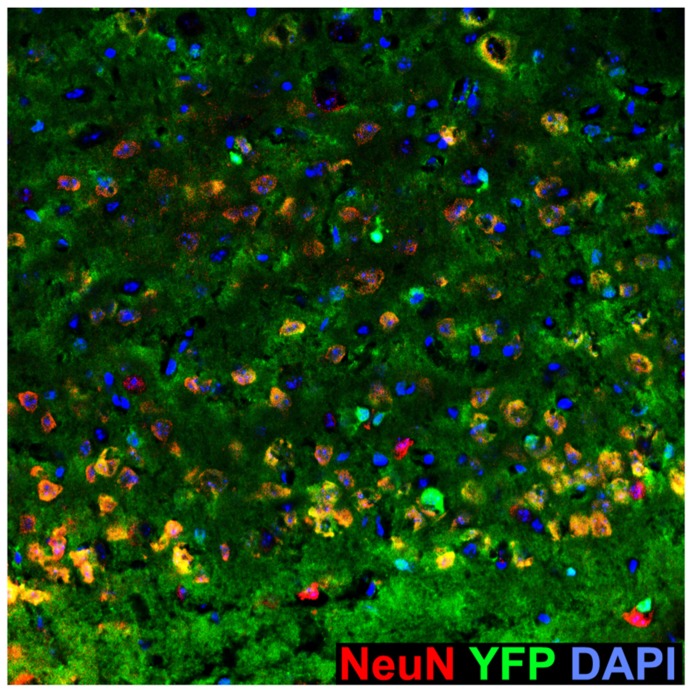
**Abundant YFP+ cells in the *Emx1-CreRosa26***^YFP^/^+^ motor cortex. Sagittal section of the *Emx1-Cre Rosa26*^YFP/^^+^ motor cortex immunostained for YFP and the neuronal marker NeuN, which labels all cortical neurons. Note the presence of NeuN–, YFP+ cells, likely corresponding to glia.

Territories positive for EGFP-VAMP2 in *Emx1-Cre*+* Rosa26^EGFP-VAMP2/+^* mice were also analyzed by immunohistochemistry on brainstem and spinal cord sections. At the origin of the pyramidal tract, in the cerebral hemispheres, EGFP decorates cell-poor, fiber rich regions (**Figure A2A**). In the medulla oblongata (**Figure A2B**, inset magnified in C), where all cells are *Emx1* negative, the pyramids, containing descending corticospinal fibers of passage, were weakly positive. Instead, strongly labeled, EGFP-positive, presumptive presynaptic puncta were detected in pontine nuclei of *Emx1-Cre*+* Rosa26^EGFP-VAMP2/+^*mice. In particular, punctate signal was detected at the location of basal pontine nuclei with a non-cellular pattern (**Figures 4D–F**). The distribution was similar to the one reported in the literature for cortico-pontine terminals using anterograde tracers (Bjaalie et al., 2005). EGFP immunoreactive terminals colocalized with the pan-vesicular marker synaptotagmin-1 (Syt-1, **Figure 4**). Taken together, these data suggest that our reporter does not label cell bodies, stains axons weakly, and decorates axon terminals more strongly, as expected of a properly sorted synaptic vesicle marker.

### MOST *EGFP-VAMP2*+* CST* TERMINALS DECORATE DORSAL AND INTERMEDIATE LAMINAE IN THE SPINAL CORD GRAY MATTER

**FIGURE 5:**
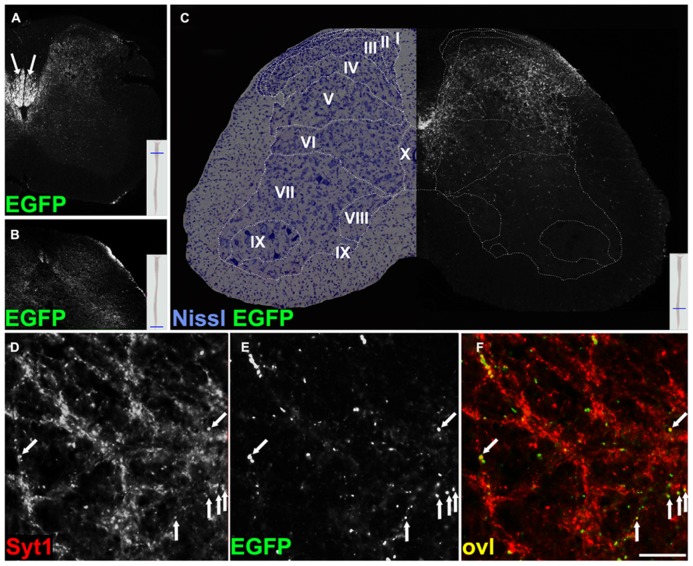
**EGFP decorates presynaptic terminals in the spinal cord of *Emx1-Cre Rosa26^EGFP-VAMP2/+^* mice and colocalizes with synaptotagmin 1 positive puncta.** Transverse sections of the spinal cord at cervical **(A)**, sacral **(B)**, and lumbar **(C)** levels. In **(A)**, strong signal is seen in the dorsal white matter (arrows; see text for discussion). In **(C)**, a Nissl-stained lumbar cord hemisection is juxtapposed to its adjacent section, stained for EGFP. Stippled lines delimit Rexed laminae (I–IX). Note that the majority of corticospinal terminals are located in dorsal and intermediate laminae, while few occupy lamina IX, containing the bodies of spinal motoneurons*.* (**D**–**F)** Transverse sections of the spinal cord gray matter at the lumbar level. EGFP signal from corticospinal axon terminals in **(B)** colocalizes with synaptotagmin 1 (Syt1) in **(A)**. Overlay (ovl) in **C** (arrows). Scale bar for **(D**–**F)** 25 μm.

Next, we focused on the analysis of CST terminals. In the spinal cord of *Emx1-Cre*+* Rosa26^EGFP-VAMP2/+^*mice, immunoreactivity for the fusion protein spanned cervical (**Figure 5A**) through sacral (**Figure 5B**) segments. More precisely, we found strongly labeled axons primarily in the funiculi of the dorsal column, where mouse CST axons descend after decussating in the medulla (Steward et al., 2004; Bareyre et al., 2005 and others). Obviously, the more caudal the segment, the less signal we detected in the white matter, due to the progressive depletion of corticospinal axons. However, the most remarkable feature was the punctate pattern clearly visible in the gray matter, matching the distribution of corticospinal synaptic terminals. To further characterize where corticospinal axons terminate, we analyzed the cytoarchitecture of a lumbar section immediately adjacent to the one stained by immunofluorescence, and based our analysis on segment-specific Rexed lamination (**Figure 5C**). The strongest signal was confined to the dorsal horn and to the zona intermedia, mainly in laminae III-VI, with only sparse immunoreactive *puncta* in laminae VII-IX. These data are in close agreement with published results obtained using anterograde tracing (Kuypers and Martin, 1982; Holstege, 1996; Steward et al., 2004). Again, almost all EGFP+ *puncta* residing in the gray matter colocalized with syt-1 (**Figures 5D–F**).

### EVIDENCE SUGGESTING THE EXISTENCE OF LOW-FREQUENCY MONOSYNAPTIC CORTICAL CONNECTIONS ON MOTOR NEURONS AND ON RENSHAW CELLS IN MICE

**FIGURE 6:**
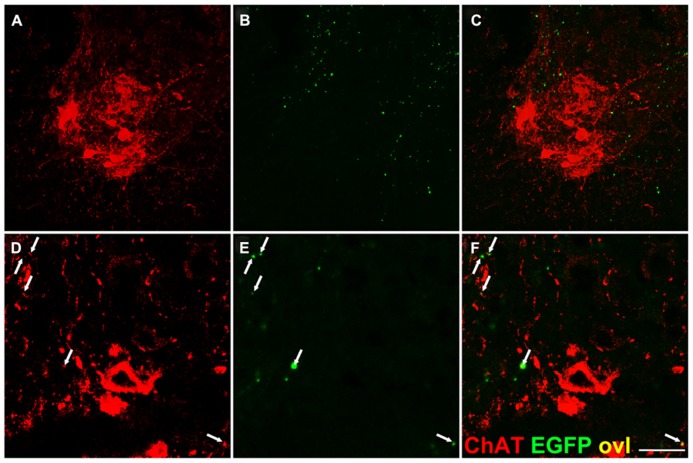
**Infrequent EGFP+ puncta in the proximity of lamina IX motor neuron somata.** Transverse sections of the spinal cord gray matter at the cervical level, lamina IX. **(A,D)** Choline acetyl transferase (ChAT) immunostaining. **(B,E)** EGFP immunostaining; **(C,F)** overlay. Very few corticospinal terminations are found on motor neuron cell bodies, while sparse EGFP+ puncta are detected on ChAT+ presumptive dendrites (arrows). Note larger size of some EGFP+ dots likely due to tyramide mediated signal amplification. Scale bar: **(A**–**C)** 75 μm; **(D**–**F)** 30 μm.

Next, we searched for evidence of direct connections between corticospinal fibers and spinal motor neurons of the ventral horns. We performed double immunofluorescence on cervical spinal cord sections of *Emx1-Cre*+* Rosa26^EGFP-VAMP2/EGFP-VAMP2^*mice immunostaining sections for EGFP and ChAT, a well-established marker of cholinergic spinal motor neurons of lamina IX. Only infrequent, scattered EGFP-VAMP+ *puncta* were observed on or flanking ChAT immunoreactive motor neurons, and they localized mainly to proximal dendrites (**Figure 6**).

**FIGURE 7:**
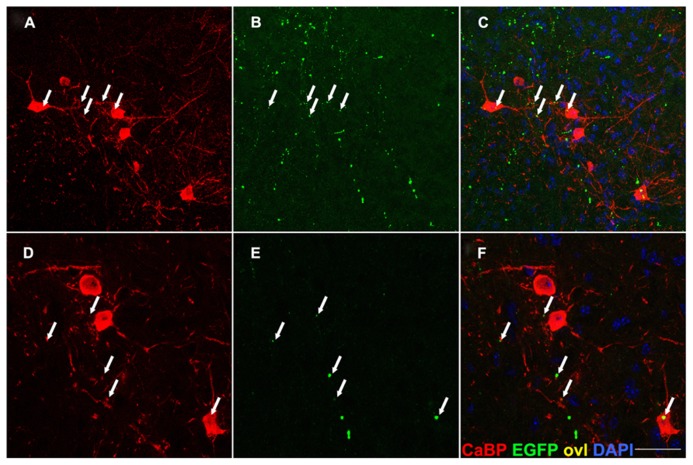
**EGFP+ presynaptic terminals are detectable on calbindin-IR neurons in lamina VII.** Transverse sections of the spinal cord gray matter at the cervical level, lamina VII. EGFP+ axon terminals (arrows in **B,E**) are detected on the soma and dendrites of calbindin (CaBP) immunoreactive cell bodies **(A,D)** likely corresponding to Renshaw neurons. Overlay in **(C,F)**. This finding suggests the previously unreported existence of CS afferents on these interneurons. Scale bar: **(A–C)** 75 μm; **(D–F)** 45 μm.

In addition, transverse spinal sections from the same Tg line were immunostained for Calbindin-D28k (CaBP), a marker of Renshaw cells, which are inhibitory interneurons residing in lamina VII of the spinal cord (Carr et al., 1998; Alvarez and Fyffe, 2007). The neuronal identity of Renshaw cells was established also based on morphology, position and size (**Figure 7**). Termination-like EGFP+ *puncta* were observed on Renshaw cell somata, or adjacent to presumptive dendrites, suggesting the previously unreported existence of likely monosynaptic connections between these glycinergic neurons and glutamatergic CST presynaptic boutons (**Figure 7**). In keeping with this observation, Renshaw cells express abundant AMPA receptors (GluR2 and 4), suggesting that they receive glutamatergic presynaptic terminals (Alvarez and Fyffe, 2007).

From this analysis, we conclude that very few corticospinal terminals effectively synapse on mouse motor neurons, suggesting that the CST plays a minor role in the control of fine movements in this species. Intriguingly, we also provide preliminary evidence of a possible direct connection between *Emx1*+ corticospinal neurons and Renshaw cells in lamina VII, which could have implications relevant to the study of normal and diseased spinal cord circuits.

## DISCUSSION

### THE *Rosa26^EGFP-VAMP2^* LINE PERMITS SELECTIVE VISUALIZATION OF PRESUMPTIVE AXON TERMINALS

In the present paper we describe a new Rosa26 knock-in line carrying an EGFP-based *Cre*-inducible presynaptic reporter. We analyzed the pattern of EGFP-immunoreactivity in three different circuits (pontocerebellar, CPT and CST) and demonstrated in all cases a highly selective localization of the reporter molecule in the putative presynaptic terminal, while axons were only detectable in the context of densely fasciculated white matter tracts such as the dorsal funiculus. While EGFP-VAMP2 did not decorate cell bodies of *Cre* positive cells in the brain cortex(**Figure A2A**) and labeled axons weakly (**Figures A2B,C**), it was spatially restricted and significantly enriched in axonal terminals (**Figures 2** and **3**); moreover, it colocalized with different presynaptic markers and labeled likely presynaptic *puncta* with the expected size and shape (**Figures 3,4** and **5**). Notably, all the targets of these tracts were globally *Cre* negative (with the exception of the few *Syn1-Cre*+ GABAergic interneurons in the GL of the cerebellar cortex, (Figure A1). Significantly, reporter targeting was highly efficient even in very long range axonal tracts, such as the CST (**Figures 5–7**), whose length in the mouse exceeds the diameter of pyramidal neuron bodies by three orders of magnitude.

### EVIDENCE OF CORTICOSPINAL TERMINATIONS ON SPINAL MOTORNEURONS

The *Rosa26^EGFP-VAMP2/+^*line was used in this work to tackle a controversial issue: whether or not CS terminals establish direct connections with spinal motor neurons in the mouse (**Figure 6**). To date, neuroanatomists disagree in regard to the contribution of the corticospinal pathway on motor function in species other than primates. The CST is a composite, species-specific pathway; it has several functions and originates from a variety of cortical areas, including classical cortical motor areas (as the primary motor cortex, the premotor cortex, the supplementary motor area), the anterior cingulate cortex (Dum and Strick, 1996) and even sensorimotor areas, as the somatosensory cortex, the parietal operculum and the posterior parietal cortex (Kuypers and Martin, 1982). Fibers coming from each area will eventually synapse in the spinal cord in a discrete fashion: sensorimotor inputs preferentially terminate in the dorsal horn, while motor fibers ultimately synapse in the zona intermedia and, to a lesser extent, in the ventral horn (Dum and Strick, 1996; Lemon and Griffiths, 2005; Lemon, 2008). Ninety percent of the murine pyramidal tract is made up of sensorimotor projections, which terminate in laminae II-V (Steward et al., 2004; Bareyre et al., 2005; Lemon and Griffiths, 2005; Lemon, 2008); accordingly, our results show an accumulation of CST terminations in the spinal dorsal horn (**Figure 5C**).

Early observations made in rats by neuroanatomical tracing using low resolution first generation methods (Casale et al., 1988) have led neuroanatomists to the conclusion that CS terminals are fairly abundant in laminae III – VI, and sparse in lamina VII, while no direct monosynaptic connections were reported in lamina IX. Likewise, attempts to elicit sustained excitatory postsynaptic potentials (EPSP) in motor neurons through electrical stimulation of the CST have been unsuccessful (Alstermark et al., 2004). Other neuroanatomical studies have also led to the conclusion that the CST only plays a minor role in the initiation of limb movement (Lemon, 2008). At odds with these conclusions, corticospinal terminations have been detected by other authors on rat (Bareyre et al., 2002), mouse (Bareyre et al., 2005) and hamster (Kuang and Kalil, 1990) motor neurons. Contradictory results have emerged from electron microscopy studies as well (Curfs et al., 1996; Yang and Lemon, 2003). Our genetic evidence adds to the results of previous investigations, hopefully contributing to the establishment of a consensus in regard to this highly debated topic. Our evidence speaks for the existence of only a small number of direct corticospinal connections on the surface of spinal motor neurons. In comparison to previously used genetic reporters, the *Rosa26^EGFP-VAMP2^* model features an increased selectivity and is significantly more efficient in lighting up presumptive presynaptic terminals than axon shafts.

Taken together, our results are consistent with the notion that while scattered monosynaptic connections exist in mice, most functional motor tasks are controlled through polysynaptic pathways in this species. It is now widely accepted that synaptic contacts and alterations may influence at least in part the progression of neurodegenerative diseases (reviewed in Conforti et al., 2007). ALS primarily involves the upstream and downstream halves of a monosynaptic circuit, including the pyramidal neuron of cortical layer 5 and the spinal motor neuron. If such a circuit is indeed less crucial in mice than in humans, then the results stemming from murine models of ALS should be interpreted cautiously, especially as regards CST involvement in this disease.

### EVIDENCE FOR THE EXISTENCE OF LIKELY DIRECT CONNECTIONS BETWEEN THE PYRAMIDAL TRACT AND RENSHAW CELLS

The *Rosa26^EGFP-VAMP2^* mouse strain has also made it possible to visualize putative direct connections from the cortex to Renshaw cells (**Figure 7**), glycinergic neurons that receive antidromic signals from spinal motor neurons. To the best of our knowledge, this represents an entirely novel finding. Intriguingly, *Emx1* is expressed by glutamatergic neurons, suggesting that a subset of CS fibers carries excitatory stimuli to Renshaw cells. It is well established that Renshaw cell axons make local arborizations but also extend to other ipsilateral spinal segments (Alvarez and Fyffe, 2007). Thus, through a direct corticospinal connection with Renshaw cells, a pool of spinal motor neurons may be subjected to an inhibitory, inter-segmental control. If this were the case, it may have important implications for motor control in physiology and disease. Further studies are required to gauge the pathophysiological relevance of these observations.

## Conflict of Interest Statement

The authors declare that the research was conducted in the absence of any commercial or financial relationships that could be construed as a potential conflict of interest.
